# Differences in Three-Dimensional Geometric Recognition by Non-Cancerous and Cancerous Epithelial Cells on Microgroove-Based Topography

**DOI:** 10.1038/s41598-017-03779-6

**Published:** 2017-06-26

**Authors:** Keiichiro Kushiro, Tomohiro Yaginuma, Akihide Ryo, Madoka Takai

**Affiliations:** 1 Department of Bioengineering, School of Engineering, The University of Tokyo, 7-3-1 Hongo, Bunkyo-ku, Tokyo 133-8656 Japan; 2 Department of Microbiology, School of Medicine, Yokohama City University, 3-9 Fukuura, Kanazawa-ku, Yokohama 236-0004 Japan

## Abstract

During metastasis, cancer cells are exposed to various three-dimensional microstructures within the body, but the relationship between cancer migration and three-dimensional geometry remain largely unclear. Here, such geometric effects on cancerous cells were investigated by characterizing the motility of various cancer cell types on microgroove-based topographies made of polydimethylsiloxane (PDMS), with particular emphasis on distinguishing cancerous and non-cancerous epithelial cells, as well as understanding the underlying mechanism behind such differences. The 90-degree walls enhanced motility for all cell lines, but the degrees of enhancements were less pronounced for the cancerous cells. Interestingly, while the non-cancerous epithelial cell types conformed to the three-dimensional geometrical cues and migrated along the walls, the cancerous cell types exhibited a unique behavior of climbing upright walls, and this was associated with the inability to form stable, polarized actin cytoskeleton along the walls of the microgrooves. Furthermore, when non-cancerous epithelial cell lines were altered to different levels of polarization capabilities and cancer malignancy or treated with inhibitory drugs, their three-dimensional geometry-dependent motility approached those of cancerous cell lines. Overall, the results suggest that cancerous cells may gradually lose geometrical recognition with increasing cancer malignancy, allowing them to roam freely ignoring three-dimensional geometrical cues during metastasis.

## Introduction

Cancer metastasis, which is a cell migration phenomenon through the various microstructures in the body, often leads to fatal secondary tumors, and this phenomenon has been recently reported to be influenced by the surrounding microenvironments around the tumor^1–3^. For example, breast tumor cells have been shown to rearrange the surrounding extracellular matrix (ECM) by aligning and bundling collagen fibers or by forming cell-sized, tube-like microtracks to facilitate metastasis^4–7^. However, how the microstructures affect the metastatic capabilities of cancer cells and the underlying mechanotransduction mechanisms still remain largely unclear.

In general, the migration machinery and mechanotransduction of cancer cells are equivalent to the non-cancerous counterparts^8–13^. However, cancer cells exhibit high plasticity in terms of invasion mechanisms^14–16^, and as such, the cancer migration is a complicated phenomenon which is altered by various signaling molecules and environmental factors, including their three-dimensional physical surroundings. In order to better understand the cancer migration behaviors and the underlying mechanisms, researchers have resorted to micro- and nano-fabrication methodologies to create simplistic, *in vitro* model platforms. In the past, surface structure (topography) and microchannel systems have been shown to influence the polarization and cell motility behaviors^17–19^. In particular, cancer cells on various sub-cellular microtopography or in various microchannel structures have been shown to enhance migration speed, and some suggested that perhaps the mode of migration may have shifted towards amoeboid-like migration^20–22^. Nonetheless, the purely geometrical influence of the microstructures and the underlying mechanotransduction largely remain unresolved, so there is a need for a more comprehensive study on how the different types of cancer cells, especially those with the same lineage but differing levels of malignancy, respond to microstructures, and how such behaviors may differ from non-cancerous cells.

In recent years, we reported on the cell motility enhancement phenomenon using microgroove-based structures of specific geometrical parameters, in which non-cancerous epithelial cells were shown to drastically alter their morphology and motility when they come in contact with the walls of such structures^23^. In this study, using such alterations to cell motility caused by topography as a quantitative measure of the degree of structural recognition, we investigated the interactions between various epithelial cancer cell lines of differing types and malignancies and microgroove-based structures. The aim of this research was to deepen our knowledge of the topographical influence on cancer migration behaviors and the underlying mechanotransduction mechanisms, to not only better understand cancer metastasis, but also to serve as a stepping stone for designing topography-based biodevices that may isolate and characterize epithelial cancer cells based on the actual migratory behaviors, as opposed to the traditional membrane surface markers.

## Results and Discussion

### Specific geometry of microgroove structures cause cell motility enhancements and unique behaviors from cancerous cell types

**Figure 1:**
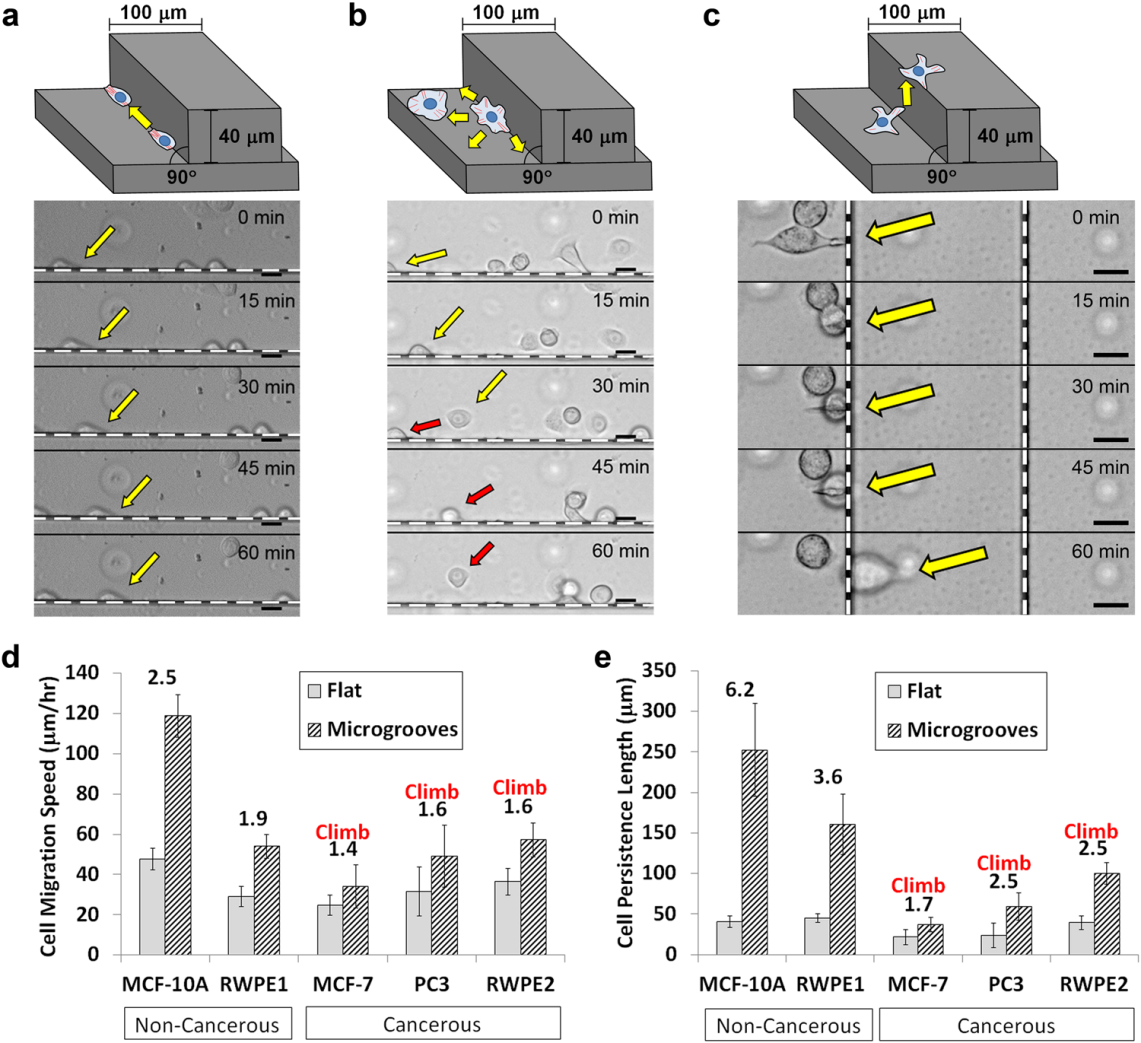
Schematic illustrations of the different modes of cell migration on the PDMS microgroove structures and time-lapse images of (**a**) RWPE1 (non-cancerous; persistent) and (**b**) RWPE2 (cancerous; non-persistent) cell migration along the groove walls (the dotted white lines in each image), as well as (**c**) PC3 (cancerous) cell climbing over the walls. Colored arrows point to notable cells in each image. (Scale bar, 20 μm) (**d**) Speed and (**e**) persistence length measurements of various non-cancerous and cancerous epithelial cell types on the flat surfaces and at the corners of the microgrooves. Numbers above the bars represent the quantitative effect of topography as the ratio between the groove and flat measurements. “Climb” signifies that the cell type could climb over 40-µm-high walls. Error bars are SEM (N = 5 trials with 10–20 cells quantified per trial).

Various non-cancerous and cancerous cell types were seeded on PDMS microgroove structures. Majority of the cell lines consisted of breast and prostate epithelial cell lines that are non-cancerous (MCF-10A and RWPE1) and cancerous (MCF-7, RWPE2 and PC3). Other cell types such as lung epithelial cells (H292), fibroblasts (L929) and macrophages derived from THP-1 leukemic monocytes (THP-1) were also tested for comparison as well (Supplementary Fig. S1). There were significant behavioral differences in the cell movements between cancerous and non-cancerous cells on the microgroove substrates (Supplementary Videos S1–S5). The non-cancerous cells often traveled unidirectionally along the walls of the microgroove structures (Fig. 1a), resulting in high persistence, similar to the behaviors seen in the previous study^23^. Meanwhile, the cancerous cells often changed directions or detached from the walls (Fig. 1b), regardless of the degree of speed or speed enhancements. Interestingly, all cancerous cells tested were able to vertically climb the 40-µm-high walls of the microgroove structures (Fig. 1c), a behavior not observed in any of the non-cancerous cells. The rate of wall climbing were dependent on the cell types, with the THP-1 immune cells being the fastest, PC3 and RWPE2 cells in the middle with similar speeds, and MCF-7 cells being the slowest among the group (Supplementary Videos S3–S5). The quantitative measurements for the cell migration speeds and persistence lengths (distance traveled before changing directions) for the various cell types are summarized in Fig. 1d and e, respectively. It was found that the microgroove structures enhanced the speeds and persistence lengths of all cell types, regardless of being cancerous or not. Such topographical enhancements were characterized by taking the ratios of the motility parameters from the flat surface and the microgroove measurements, which also serves as a quantitative measure of the degree of structural recognition. The topographical effects on the persistence length were more prominent in non-cancerous cells as compared to their cancerous counterparts (*e.g*., MCF-10A and MCF-7; RWPE1 and RWPE2), suggesting a reduced topographical recognition by the cancerous cell types. Also, the innately motile cells tended to exhibit a high degree of motility enhancement (>1.7 for speed) from the topography effect (*e.g*., MCF-10A and THP-1), while those that appeared extensively spread and had low base motility (*e.g*., L929, H292) generally exhibited a low degree of motility enhancement (<1.3 for speed).

**Figure 2:**
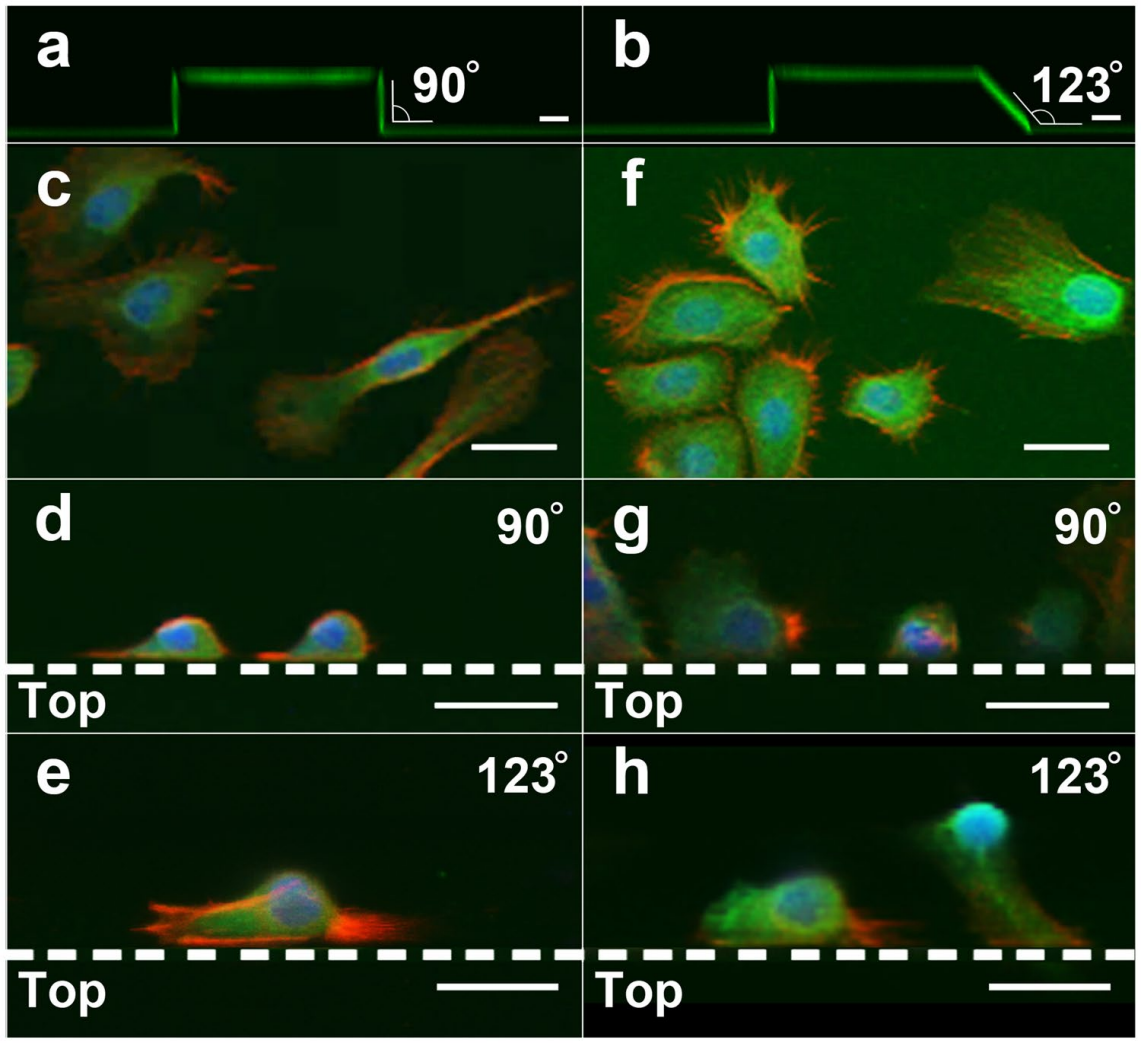
Confocal image of the microgroove-based structures with (**a**) 90° wall and (**b**) 123° wall visualized by the adsorbed FITC-BSA. Immunostaining (20x) of actin (red), vinculin (green) and nucleus (blue) of (**c–e**) RWPE1 (non-cancerous; polarized) and (**f–h**) RWPE2 (cancerous; non-polarized) cells on (**c,f**) flat surfaces, (**d,g**) upright microgroove structures and (**e,h**) slanted microgroove structures. Dotted lines represent the microgroove wall boundaries, and the ‘Top’ area below the lines represent the top plateau of the microgroove structures. (Scale bar, 20 μm).

Furthermore, the influence of the geometrical parameters on the epithelial cells were investigated. First, the angles of the walls were altered between 90 degrees and 123 degrees, as confirmed by the fluorescence visualization of the adsorbed proteins on the surface by confocal microscopy (Fig. 2a and b). Similar to our previous results^23^, the normal RWPE1 cells displayed reduced topography-induced motility enhancements as the angle of the microgroove walls became more obtuse, but the cancerous RWPE2 cells were less influenced by the changes in the wall angle in terms of their motility and the wall climbing capabilities (Supplementary Fig. S2). Again, the results suggest that the cancerous cells are less sensitive to the physical cues provided by the microgroove structures. In addition, climbing of walls higher than 100 µm by individual cancer cells was not observed, while even non-cancerous cells could climb the walls if the wall height was below 20 µm (data not shown). Thus, the distinction between the capabilities of cancerous and non-cancerous cells to climb walls depended on the specific height of the microgroove structures.

### Immunostaining of cancerous and non-cancerous epithelial cell types and the roles of actin organization and focal adhesions

**Figure 3:**
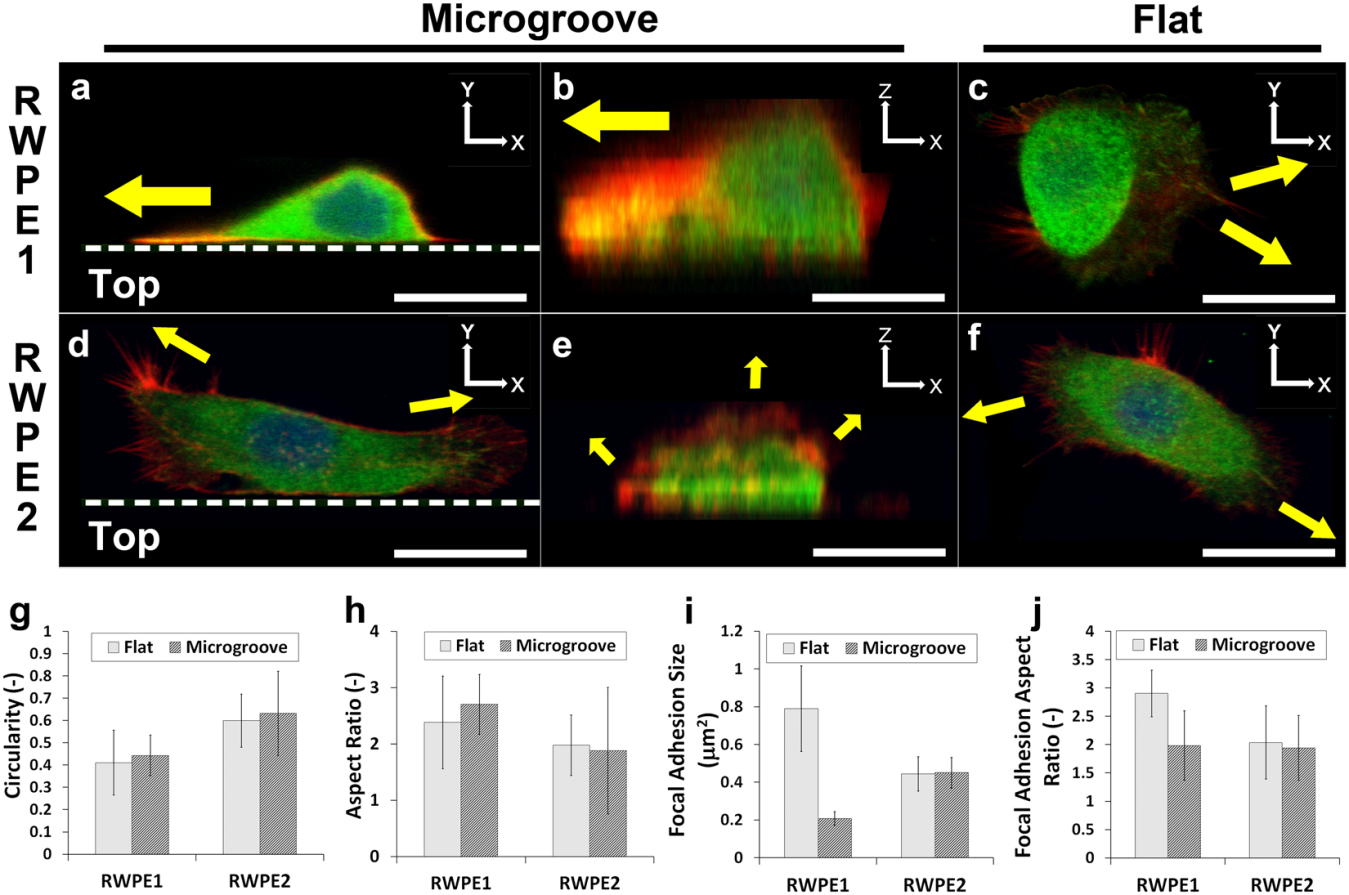
Immunostaining (63x) of actin (red), vinculin (green) and nucleus (blue) of (**a–c**) RWPE1 (non-cancerous; polarized) and (**d–f**) RWPE2 (cancerous; non-polarized) cells on (**c,f**) flat surfaces and on microgroove structures, with the same cell visualized on (**a,d**) the X-Y plane and (**b,e**) the Z-X plane. Dotted lines represent the microgroove wall boundaries, and the ‘Top’ area below the lines represent the top plateau of the microgroove structures. Yellow arrows indicate the directions of the major actin extensions. (Scale bar, 20 μm) (**g**) Circularity and (**h**) aspect ratio for RWPE1 and RWPE2 cells on flat surfaces and microgroove structures. (**i**) Focal adhesion size and (**j**) focal adhesion aspect ratio for RWPE1 and RWPE2 cells on flat surfaces and microgroove structures. Error bars are SEM (N = 3 trials with 20 cells quantified per trial).

In order to understand the mechanisms underlying such motility differences, immunostainings for cytoskeletal components of the cells, such as actin stress fibers and focal adhesion molecule vinculin, were performed. The results showed that the motile, non-cancerous RWPE1 cells were able to directionally polarize on flat surfaces (Fig. 2c) and on the microgroove walls (Fig. 2d), consistent with the high persistence observed. In addition, on the sloped microgroove structure, the cells were often moderately polarized to the axis of the walls, but not to the extent of the upright walls (Fig. 2e). On the other hand, the cancerous RWPE2 cells were less polarized on all substrates (Fig. 2f–h) with lamellipodia and invadopodia-like structures facing multiple directions, resembling morphologies observed in previous studies^24^. In addition, upon closer examination at higher magnification (63x), it was confirmed that the RWPE1 cells in contact with the microgroove structure displayed a directionally oriented stress fiber and focal adhesion distributions, where the actin filaments and vinculins were aligned against the wall and concentrated in the leading edge (Fig. 3a and b). Furthermore, the focal adhesion formations seemed to be less prominent compared to those on flat surfaces (Fig. 3c). In stark contrast, the RWPE2 cells were less directionally oriented, regardless of being in contact with the microgroove structure or not (Fig. 3d–f). Even on the plane of the wall (Z-X plane), the actin filaments were facing multiple directions, including the vertical direction (Fig. 3e). Thus, the lack of alignment to the microtopography, suggesting the lack of geometrical recognition by these cancerous cells, likely enable them to extend their protrusions in many directions and climb over the walls.

Quantitative image analyses were also performed to confirm the polarization of cells and gain insights into the mechanotransduction within these epithelial cells. The results of the circularity (Fig. 3g) and the aspect ratio (Fig. 3h) suggest that, on average, the non-cancerous RWPE1 cells are indeed more polarized than the cancerous counterpart. Furthermore, it was found that the focal adhesion size (Fig. 3i) and polarization (Fig. 3j) were dramatically affected for the RWPE1 cells upon contact with the microgroove structures. On flat surfaces, the focal adhesions of non-cancerous cells seem to undergo the traditional maturation process^13^, forming large, polarized focal adhesions, while on the microgroove walls, the mature focal adhesions were not observed. The result suggests a rapid turnover of focal adhesions, which may be culminating in the topography-induced speed enhancement. Meanwhile, for the cancerous RWPE2 cells, the formation of focal adhesions seem to be unaffected by topography. All in all, the immunostaining results suggest key roles of actin alignment, cell polarization and focal adhesion formations in controlling the motility-enhanced morphology acquired from certain microtopographies.

### Polarization capabilities and degree of cancer malignancy alter the geometrical recognition of epithelial cancer cells

To further elucidate the relationship between the cancer-associated depolarization and the effect of topography, genetic variants of MCF-10A epithelial cell with different degrees of cancer malignancy were utilized: MCF-10A APC−/− (APC knockout) and MCF-10A RAS (H-ras G12V transformed; tumorigenic). APC−/− lacks the gene for a key tumor suppressor adenomatous polyposis poli (APC), which is known to interact with Rac1 and Cdc42 through IQ-motif-containing GTPase activation protein 1 (IQGAP1) and the microtubule-stabilizing protein CLIP-170^25^. RAS is an oncogene deeply involved in the growth factor signaling pathway (Ras-Raf-MAPK) and also interferes with the cytoskeletal organization via PI3K^26^. These MCF-10A variants were also compared to the representative breast cancer cell lines, MCF-7 and MDA-MB231. MCF-7 is known to be estrogen receptor (ER) and progesterone receptor (PR) positive, while MDA-MB231 is known to be triple-negative, which lacks the expression of ER, PR and HER, and has high metastatic capabilities^27^.

**Figure 4:**
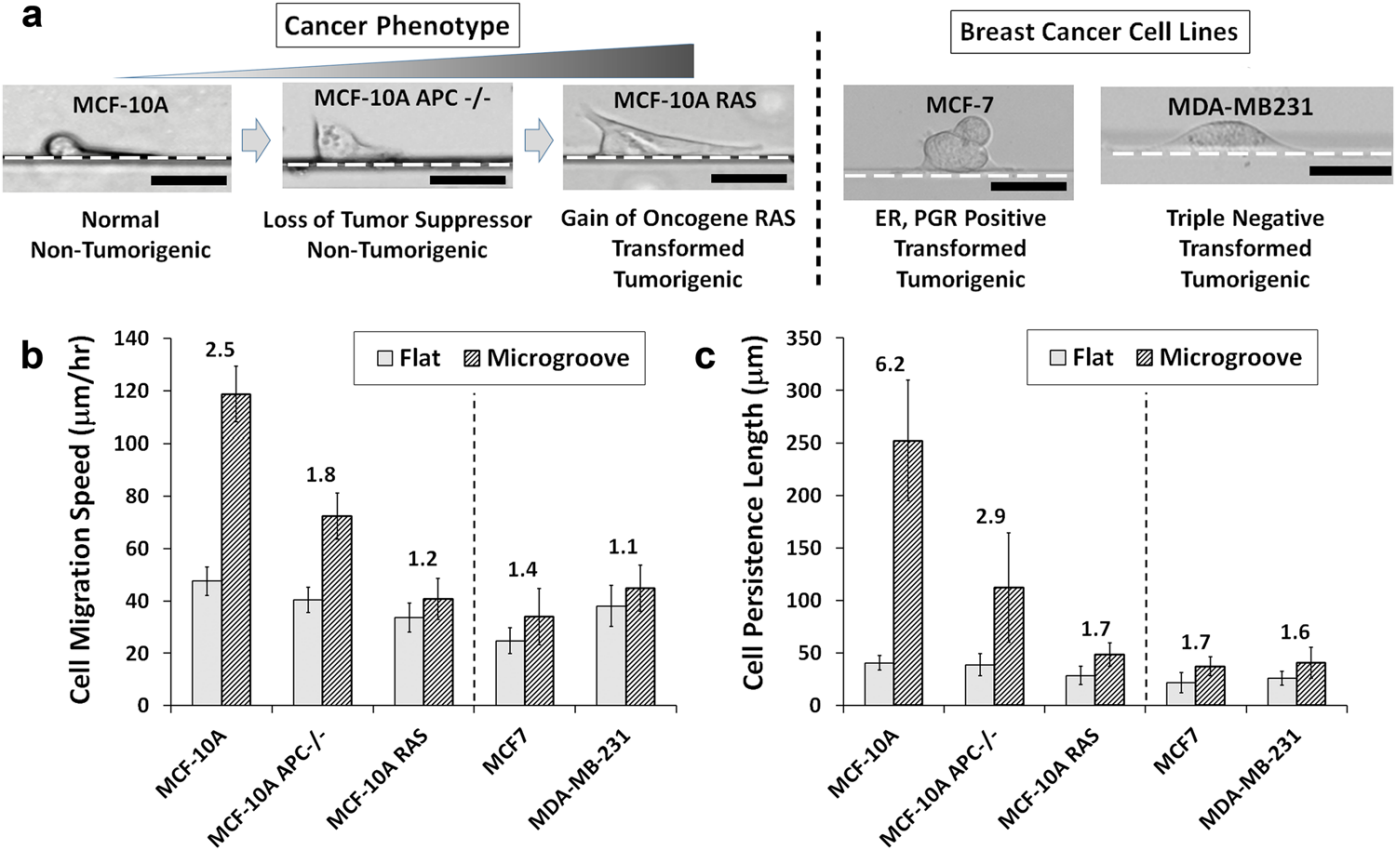
(**a**) Bright-field images of the disruption in the stable morphology with genetic alterations to the MCF-10A cells and morphologies of other breast tumor cell lines at the corners of the microgroove structures. Dotted lines represent the microgroove wall boundaries. (Scale bar, 20 μm) (**b**) Speed and (**c**) persistence length measurements of MCF-10A variants and other breast tumor cell lines on the flat surfaces and at the corners of the microgrooves. Numbers above the bars represent the quantitative effect of topography as the ratio between the groove and flat measurements. Error bars are SEM (N = 5 trials with 10–20 cells quantified per trial).

When these cell lines were tested on the microgroove structures, the stable, unidirectional morphology of the wild-type MCF-10A cells gradually deteriorated with cancer malignancy, where the formation of multiple lamellas gradually turned the highly-motile morphology into the multi-lamellar, mesenchymal morphology (Fig. 4a). Also, even though their bare motilities were comparable to the wild-type MCF-10A, the motility enhancements from the microstructures were drastically reduced in accordance with their cancer malignancy (Fig. 4b and c; Supplementary Videos S1, S6–S8). It is also noteworthy that the degree of focal adhesion strengths varied greatly among these cell types as well (Supplementary Fig. S3), suggesting that the key factor in sensing the topographical effects may be through the actin organization, more so than the integrin-mediated adhesions to the surface. In order to verify the necessity of actin stress fibers in the topography-associated motility, the non-cancerous MCF-10A cells were treated with 5 μM latrunculin A to disrupt actin polymerization. Latrunculin A treatment immediately halted cell migration along the microgroove topography with almost no alteration to the compact morphology, while gradually disrupting actin organization for the cells on flat surfaces (Supplementary Fig. S4). These results suggest that the membrane protrusion by the actin polymerization is a key driving force in topography-directed migration and that the genetic alterations that hinder the polarization mechanism can remove important factors for sensing topographical cues and gaining the motility enhancement effects from microstructures. This point was further corroborated by treating the non-cancerous MCF-10A cells with 5 μM blebbistatin to disrupt the myosin II contractility, which are known to cause the loss of polarization^28^. With the partial loss of contractility, the cells lost the unidirectional morphology and tended to aggregate more. Interestingly, the MCF-10A cells treated with blebbistatin formed more isotropic lamella and were also able to extend over the 40-µm-high walls of the microgroove structures (Supplementary Fig. S5). Perhaps the mechanotransduction sensing of the cells may have been also altered by the treatment, but this remains to be further investigated.

### Cancer-microstructure interactions and relevance to metastasis

**Figure 5:**
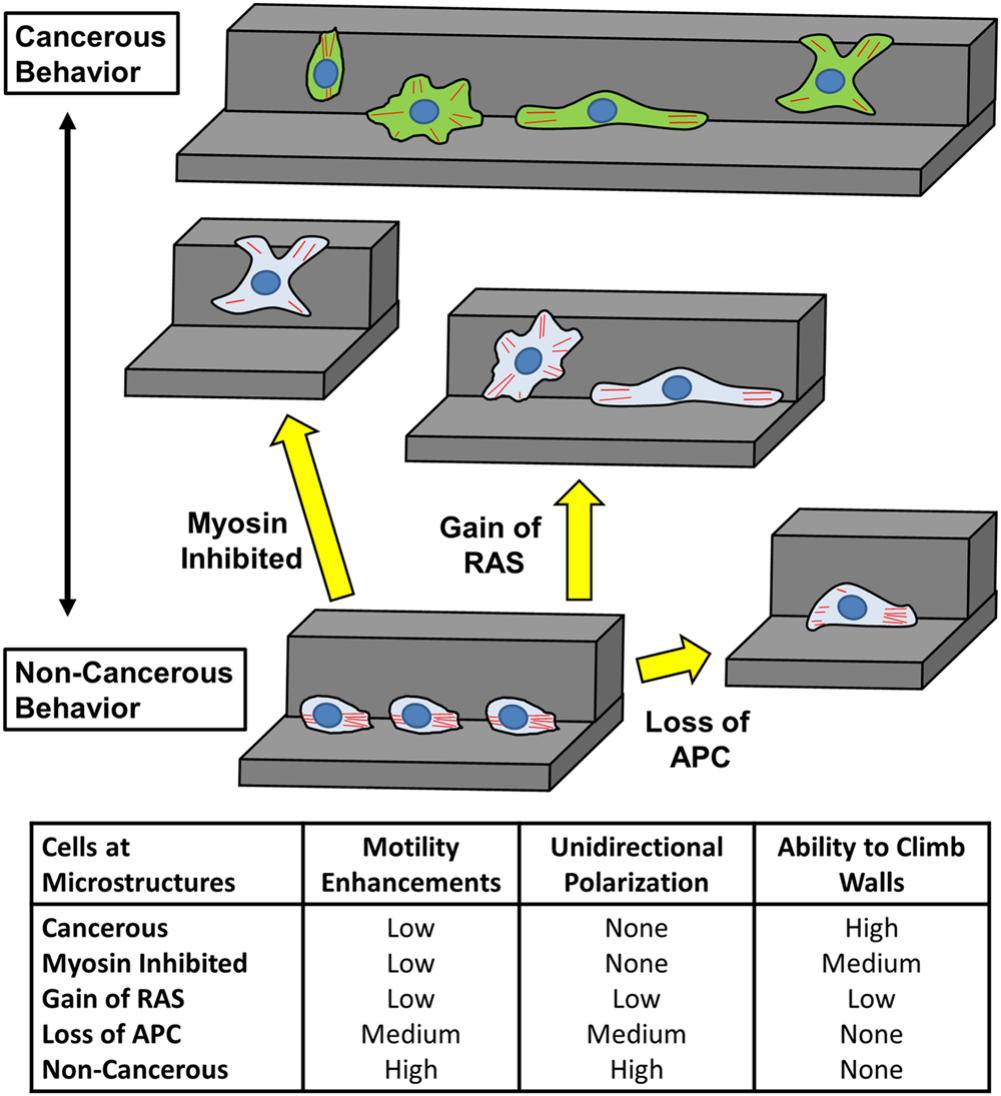
Schematic summary of the cell-geometry interactions for various cancerous and non-cancerous epithelial cells.

Through this study, it was shown that the geometrical cues from microstructure can enhance the motility of the cancer cells, consistent with some of the previous studies that suggest the reorganization of the microenvironment can facilitate the rate of cancer metastasis^29–31^. However, the prime characteristic of epithelial cancer cells was their inability to form stable and oriented actin fibers along the microgroove structures, especially with increasing cancer malignancy, which resulted in the reduced topographical enhancements in motility compared to non-cancerous cells. Furthermore, disrupting the actin organization in non-cancerous cells by knocking out APC or introducing RAS resulted in a more cancer-like behavior. Such inability to organize and polarize the cytoskeleton has also been well-documented in the medical field in the form of disorganized cancer tissues^32, 33^. Nevertheless, due to such inability to recognize the geometrical cues, these cancer cells were able to extend their lamellipodia in various directions, including over the walls of microgroove structures to climb these walls. Such behavioral insensitivity to microstructure has also been recently suggested for cell proliferation^34^. This inability to recognize geometrical cues have important implications for how the tumor cells metastasize in the body, since it suggests that as they become malignant, they may begin to ignore geometrical cues that are generally used in the body as guidances to maintain organization^35^, and thus bestow upon them the capability to roam absolutely freely in the body. These findings have been summarized in Fig. 5.

The results from these studies also resonate with some previous research that utilize microstructures to study cancer cell behaviors. Some have demonstrated that cancerous cells have increased tendencies to navigate through narrow channels with enhanced speed^21, 22, 36^, which is consistent with the results from this study where the cells are insensitive to the mechanical cues of the microstructures. In another study, researchers have shown that cancer cells are insensitive to the physical cell-cell collisions, thereby allowing metastatic cells to effectively slide by other cells and thus migrate away from the tumor source^37^. Similar tendencies were observed for highly cancerous cells migrating along the microgroove walls, where they would migrate past each other rather than changing directions in response to collisions (Supplementary Videos S8). Again, this demonstrates the insensitivity of cancer cells to the mechanical signals arising from physical surroundings.

### Concluding Remarks

In conclusion, findings in this study point to important differences in topography recognition between cancerous and non-cancerous epithelial cells, as well as some of the underlying mechanotransduction mechanisms. The results suggest that the motility of both cancerous and non-cancerous cells can be affected by material surface topographies, in this case PDMS microgroove-based walls, but with varying degrees for different motility parameters. Furthermore, we found that cancerous cells are less sensitive to the various geometrical cues and could climb over sloped and upright walls of certain heights. Immunostaining suggested that the polarization and alignment of actin cytoskeleton may play a key role in differentiating the motility of cancer and non-cancerous cells on topographical structures. Lastly, when cancer malignancy was varied through genetic manipulations, it was found that the effect of topography was reduced in proportion to the cancer malignancy. These findings offer new insights into how metastatic cancer cells may ignore geometrical cues which would allow them to roam freely in the body. Also, the differences in migration behaviors among various cancerous and non-cancerous cells on these microgroove structures may be useful in characterizing the metastatic capabilities of cancer cells or separating them based on cell motility, as opposed to traditional methods that rely on antibodies for surface markers and cannot overcome the heterogeneity of the tumor population^38^. Future works to investigate the precise mechanisms behind these phenomena may lead to topography-based biodevices that can robustly and cost-effectively isolate and diagnose metastatic cells.

## Methods

### Fabrication of PDMS microgroove structure with various geometries

Microgroove structures (100-μm-wide and 40-μm-high) were fabricated using standard lithography methods by casting polydimethylsiloxane (crosslinker:elastomer ratio = 1:10) onto photoresist molds, created by spin-coating SU-8 2015 negative photoresists (MicroChem) on glass slides at 3500 rpm and exposing them to UV via the micropatterned chrome mask (Mitani Micronics). The PDMS structures were rendered cell adhesive by adsorbing fibronectin overnight (10 μg/mL). The cells were seeded on these substrates at 2 × 10^4^ cells/mL and observed for 2 days with an incubated optical microscope. For the microgroove structures with slanted walls (100-μm-wide and 40-μm-high; wall angles = 90, 105, 111, 116 and 123 degrees), the custom-made Ni mold created by Optnics Precision, Co. was used as the template^23^.

### Various cell cultures

MCF-10A human epithelial cells were cultured in growth medium composed of Dulbecco’s modified Eagle’s medium/Ham’s F-12 containing HEPES and L-glutamine (DMEM/F12, Invitrogen) supplemented with 5% horse serum (Invitrogen), 1% penicillin/streptomycin (Invitrogen), 10 µg/mL insulin (Sigma), 0.5 µg/mL hydrocortizone (Sigma), 20 ng/mL EGF (Peprotech) and 0.1 µg/mL cholera toxin (Sigma) and maintained under humidified conditions at 37 °C and 5% CO_2_. Cells were passaged regularly by dissociating confluent monolayers with 0.05% trypsin-EDTA (Invitrogen) and suspending cells in DMEM/F12 supplemented with 20% horse serum and 1% penicillin/streptomycin. Cells were passaged at 1:4 in growth medium.

Other non-cancerous cells tested were RWPE1 (epithelial) and L929 (fibroblast); cancer cells tested were MCF-7 (epithelial), RWPE2 (prostate), PC3 (prostate), H292 (lung) and THP-1(leukemia)-derived macrophages. As for the genetically modified MCF-10A variants, MCF-10A APC−/− were purchased from Sigma Aldrich, while the MCF-10A RAS was transformed via introduction of the HrasV12 oncogene^39^. They were all cultured in accordance with the ATCC recommended media and passage protocols. Also, blebbistatin (Sigma) or latrunculin A (Wako) dissolved in dimethylsulfoxide (DMSO) was used at 5 μM in growth media for the myosin or the actin polymerization inhibition experiments, respectively.

### Time-lapse microscopy and motility quantification

Cells were seeded at 2 × 10^4^ cells/mL in growth medium for 2 hr on the micropatterned substrates. After several washes to remove non-adherent cells, the culture was incubated with fresh growth medium for 4 hr and imaged at 10x magnification every 5 min using CCM-1.4II D/C Cell Culture Monitoring System (ASTEC). In this system, the cells were maintained at 37 °C and 5% CO_2_ in a heated chamber with temperature and CO_2_ controller during time-lapse imaging. CCM Software 2.4.5.2 (ASTEC) was used for image processing. To track cell motility on micropatterned surfaces, the position of the lamellipodial edge was tracked using CCM and ImageJ software. Speed was calculated as distance/time and persistence was measured as the distance for cell migration runs before they changed their direction by more than 90 degrees. The unpaired, two-tailed Student’s t-test was used for statistical analysis. Differences were considered significant at p < 0.05. All the statistical analysis was performed using corresponding functions in Microsoft Excel.

### Immunostaining of actin and vinculin

Cells were fixed on 35 mm glass-bottom dishes (Iwaki) using 4% formaldehyde solution (Sigma) for 20 min in room temperature, permeabilized using 0.2% Triton X-100 (Amersham Biosciences) for 10 min at 4 °C, blocked with 0.1% bovine serum albumin (Sigma) for 2 hr at room temperature, and incubated with dyes and antibodies. 4′,6-diamidino-2-phenylindole (DAPI; Invitrogen) was incubated for 10 min to visualize the cell nucleus, Alexa Fluor 594 phalloidin (Invitrogen) was incubated for 20 min to visualize the actin filaments, and primary anti-vinculin antibody (H-300; Santa Cruz Biotechnology) was incubated overnight then tagged with secondary Alexa Fluor 488 Goat Anti-Rabbit IgG antibody (Invitrogen) for 2 hrs to visualize the vinculin molecules. The fixed cells were visualized using a confocal microscope (LSM510; Carl Zeiss) at 20x and 63x magnifications. The image analysis of immunostaining images were performed manually using ImageJ. Circularity was calculated in a standard fashion as *f*_circ_ = 4π*A*/*P* ^2^, where *A* is the area and *P* is the perimeter of each cell. The aspect ratio was calculated as *A*_*R*_ = d_max_/d_min_, where d_max_ is the largest diameter, and d_min_ is the smallest diameter orthogonal to d_max_.

## Electronic supplementary material

Supplementary Information

Supplementary Video S1

Supplementary Video S2

Supplementary Video S3

Supplementary Video S4

Supplementary Video S5

Supplementary Video S6

Supplementary Video S7

Supplementary Video S8
