# Supplementary material for: Differences in Three-Dimensional Geometric Recognition by Non-Cancerous and Cancerous Epithelial Cells on Microgroove-Based Topography
Source: Sci Rep. 2017 Jun 26;7:4244. doi: 10.1038/s41598-017-03779-6 (PMC5484713; doi:10.1038/s41598-017-03779-6)
Supplement: Supplementary file 1 — Supplementary Information [file 41598_2017_3779_MOESM1_ESM.pdf]

Supplementary Information

Differences in Three-Dimensional Geometric Recognition by Non-Cancerous and Cancerous Epithelial Cells on Microgroove-Based Topography

Keiichiro Kushiro, Tomohiro Yaginuma, Akihide Ryo, Madoka Takai

Supplementary Figures

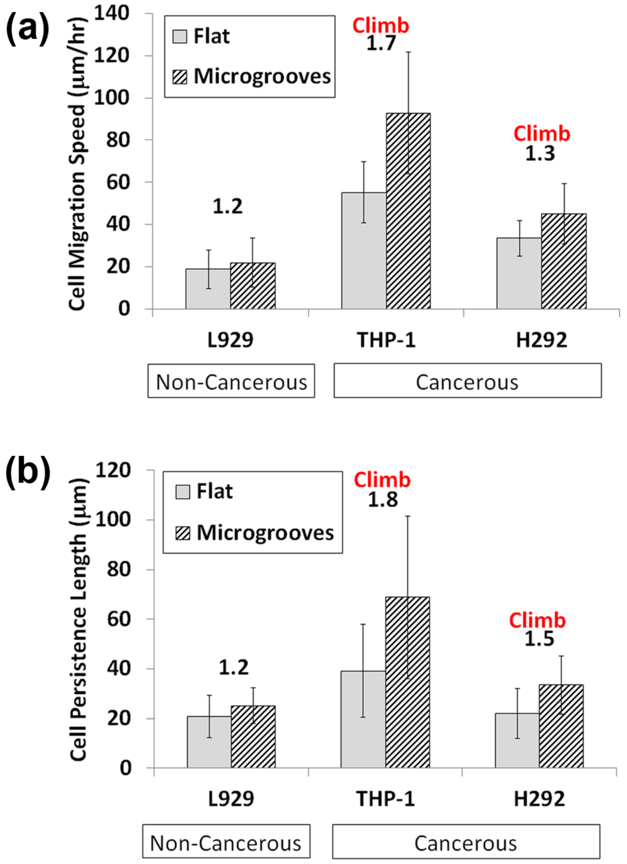

Fig. S1. (a) Speed and (b) persistence length measurements of various non-cancerous and cancerous epithelial cell types on the flat surfaces and at the corners of the microgrooves. Numbers above the bars represent the quantitative effect of topography as the ratio between the groove and flat measurements. "Climb" signifies that the cell type could climb over 40-µm-high walls. Error bars are SEM (N = 2 – 3 trials with 10 – 20 cells quantified per trial).

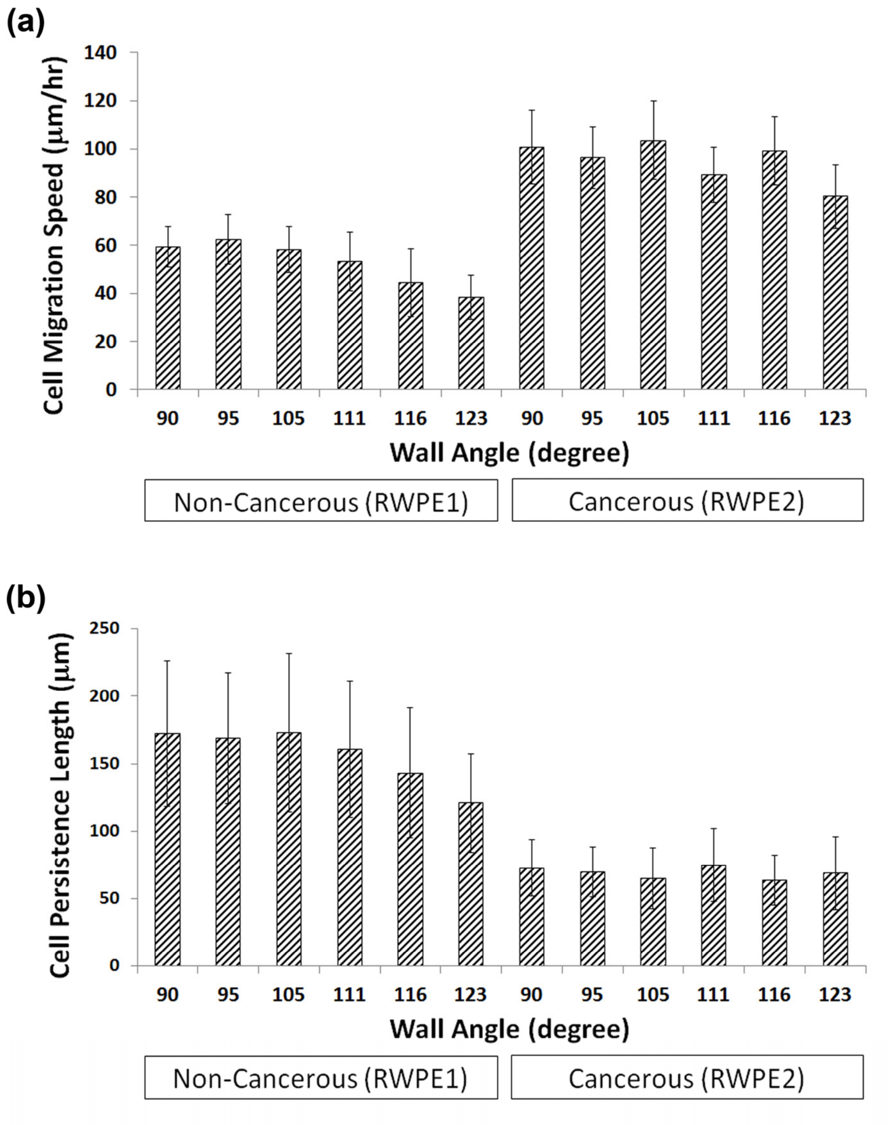

Fig. S2. (a) Speed and (b) persistence length measurements of RWPE1 and RWPE2 cells on microgroove walls of different angles. Error bars are SEM (N = 3 trials with 10 – 20 cells quantified per trial).

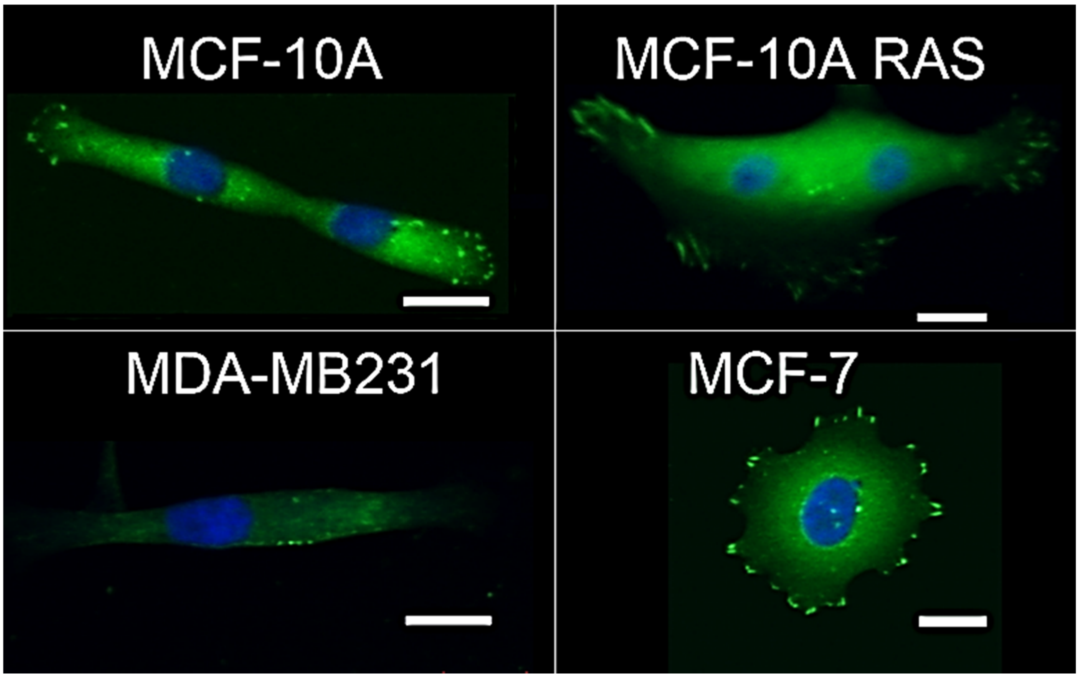

Fig. S3. Paxillin immunostaining of MCF-10A variants and other breast tumor cell lines. The MCF-10A APC-/- cell lines showed the same staining pattern as MCF-10A cells. Green = paxillin; Blue = DAPI. (Scale bar, 20 μm)

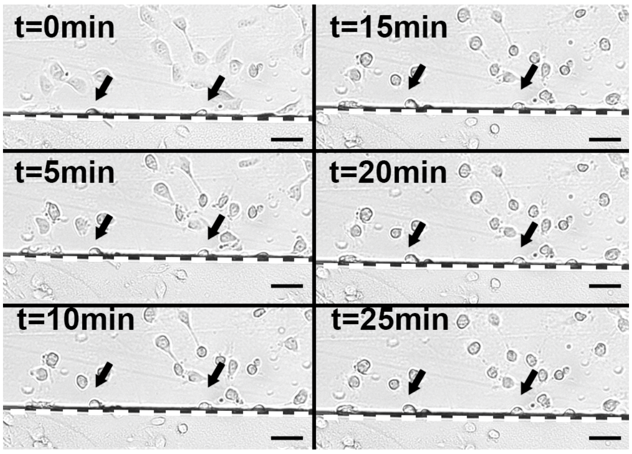

Fig. S4. The timelapse images of non-cancerous MCF-10A cells on the microgroove structures post treatment with 5 μM latrunculin A, where the drug was introduced at t = 0 min. The dotted lines

indicate the presence of the microgroove walls and the arrows point to cells of interest. (Scale bar, 20 μm)

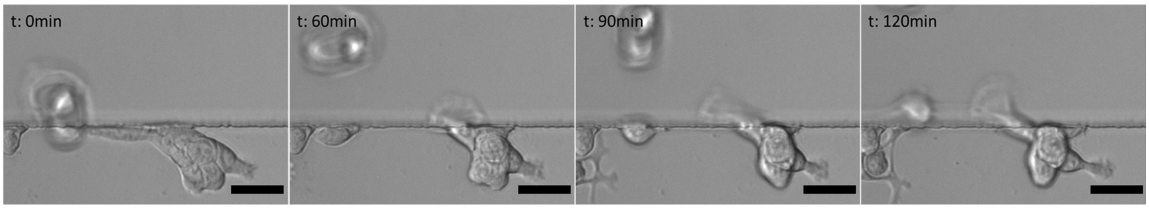

Fig. S5. The timelapse images of non-cancerous MCF-10A cells climbing the microgroove walls post treatment with 5 μM blebbistatin. (Scale bar, 20 μm)

Supplementary Videos

Video S1. MCF-10A cells migrating on PDMS microgroove topography (10x magnification). Images were acquired every 5 min for 545 min (109 frames) and compiled at 20 frames/sec in the movie.

Video S2. RWPE1 cells migrating on PDMS microgroove topography (10x magnification). Images were acquired every 5 min for 990 min (198 frames) and compiled at 20 frames/sec in the movie.

Video S3. MCF-7 cells migrating on PDMS microgroove topography (10x magnification). Images were acquired every 5 min for 855 min (171 frames) and compiled at 20 frames/sec in the movie.

Video S4. PC3 cells migrating on PDMS microgroove topography (10x magnification). Images were acquired every 5 min for 355 min (71 frames) and compiled at 20 frames/sec in the movie.

Video S5. RWPE2 cells migrating on PDMS microgroove topography (10x magnification). Images were acquired every 5 min for 660 min (132 frames) and compiled at 20 frames/sec in the movie.

Video S6. MCF-10A APC-/- cells migrating on PDMS microgroove topography (10x magnification). Images were acquired every 5 min for 1000 min (200 frames) and compiled at 20 frames/sec in the movie.

Video S7. MCF-10A RAS cells migrating on PDMS microgroove topography (10x magnification). Images were acquired every 5 min for 805 min (161 frames) and compiled at 20 frames/sec in the movie.

Video S8. MDA-MB-231 cells migrating on PDMS microgroove topography (10x magnification). Images were acquired every 5 min for 650 min (130 frames) and compiled at 20 frames/sec in the movie.
